# HER4 expression in estrogen receptor-positive breast cancer is associated with decreased sensitivity to tamoxifen treatment and reduced overall survival of postmenopausal women

**DOI:** 10.1186/s13058-018-1072-1

**Published:** 2018-11-20

**Authors:** Anja Kathrin Wege, Dominik Chittka, Stefan Buchholz, Monika Klinkhammer-Schalke, Simone Diermeier-Daucher, Florian Zeman, Olaf Ortmann, Gero Brockhoff

**Affiliations:** 1 Clinic of Gynecology and Obstetrics, University Medical Center Regensburg, Regensburg, Germany; 2 Department of Nephrology, University Hospital Regensburg, Regensburg, Germany; 3 Tumor Center Regensburg, University of Regensburg, Regensburg, Germany; 4 Center for Clinical Studies, University Hospital Regensburg, Regensburg, Germany

**Keywords:** HER4 receptor, Estrogen receptor positive breast cancer, Tamoxifen treatment

## Abstract

**Background:**

The sensitivity of estrogen receptor-positive breast cancers to tamoxifen treatment varies considerably, and the molecular mechanisms affecting the response rates are manifold. The human epidermal growth factor receptor-related receptor HER2 is known to trigger intracellular signaling cascades that modulate the activity of coregulators of the estrogen receptor which, in turn, reduces the cell sensitivity to tamoxifen treatment. However, the impact of HER2-related receptor tyrosine kinases HER1, HER3, and, in particular, HER4 on endocrine treatment is largely unknown.

**Methods:**

Here, we retrospectively evaluated the importance of HER4 expression on the outcome of tamoxifen- and aromatase inhibitor-treated estrogen receptor-positive breast cancer patients (*n* = 258). In addition, we experimentally analyzed the efficiency of tamoxifen treatment as a function of HER4 co-expression in vitro.

**Results:**

We found a significantly improved survival in tamoxifen-treated postmenopausal breast cancer patients in the absence of HER4 compared with those with pronounced HER4 expression. In accordance with this finding, the sensitivity to tamoxifen treatment of estrogen and HER4 receptor-positive ZR-75-1 breast cancer cells can be significantly enhanced by HER4 knockdown.

**Conclusion:**

We suggest an HER4/estrogen receptor interaction that impedes tamoxifen binding to the estrogen receptor and reduces treatment efficiency. Whether the sensitivity to tamoxifen treatment can be enhanced by anti-HER4 targeting needs to be prospectively evaluated.

## Background

Tamoxifen (TAM) treatment of hormone receptor-positive breast cancer (BC) has proved to be a pioneering target-specific therapy regimen [1] that was introduced more than four decades ago [2]. The main indication for an adjuvant treatment of breast cancer with anti-estrogens is the immunohistochemical identification of estrogen receptor (ER)-positive tumor cells that was suggested in 1987 [3] and has since evolved into a standardized diagnostic procedure. TAM is being frequently applied in the adjuvant (i.e., postsurgery) setting and in terms of remission maintenance and according to the treatment guidelines released by the American Society of Clinical Oncology (ASCO) and the German Gynecologic-Oncology Working Group (AGO) the application of the antiestrogen is conceived as a long-term (up to 10 years) therapy [4, 5].

From a molecular point of view TAM has been developed to competitively bind ERs located in the cell nucleus and thereby to competitively hamper the binding of estradiol. As a result, estradiol-specific (e.g., pro-proliferative) effects are inhibited and tumor growth becomes retarded or ideally even blocked [6, 7]. However, clinically short- and long-term remissions achieved by endocrine (TAM-based) treatment are often followed by the acquisition of resistance and, ultimately, disease relapse [8]. Treatment guidelines released by internationally recognized expert organizations such as the *Arbeitsgemeinschaft der Wissenschaftlichen Medizinischen Fachgesellschaften* e.V. (Berlin, Germany), the National Comprehensive Cancer Network NCCN™ (PA, USA), and the European Society for Medical Oncology (ESMO; Viganello-Lugano, Switzerland) all strongly recommend including an aromatase inhibitor (AI) into the treatment regimens for postmenopausal women (level of evidence 1b). AIs are designed to inhibit the endogenous synthesis of the estrogen receptor ligand estradiol and it has been demonstrated that a sequential targeting of the endocrine receptor ligand system prolongs the period of remission by circumventing molecular mechanism that cause treatment resistance [9, 10].

Both the ER and the human epidermal growth factor-related receptor 2 (HER2) represent dominant drivers for the genesis and progression of BC [11]. The sensitivity to target (i.e., ER and HER2 receptor) specific treatments is affected by an extensive crosstalk of receptor-triggered pathways [12]. Mechanistically, the HER2 receptor tyrosine kinase triggers a variety of downstream signaling pathways, such as the RAF/RAS/MAPK cascade that results in phosphorylation of the ER and co-regulatory molecules such as AIB1/Src-3 [12–17]. As a final consequence, TAM binding to the ER is ineffective since it does not weaken the transcriptional and pro-proliferative activity of the malignant cells. Nevertheless, HER2-induced TAM resistance is clinically manageable by switching from the anti-estrogen to an AI or by extending the treatment by an anti-HER2 targeting [18–20]. Indeed, a number of options to target alternate intracellular pathways are available to overcome HER2-induced TAM resistance [20]. However, HER2 does not work as a stand-alone receptor but forms a functional unit with its relatives HER1, HER3, and HER4. All HER receptors have prognostic impact on BC disease [21–23]; however, the extent to which HER1, HER3, and HER4 affect the sensitivity of ER-positive BC cancers to TAM treatment is hardly known. Amongst the four cognate receptor tyrosine kinases the HER4 receptor might play an exceptional role in BC biology because it has been associated either with a disadvantageous [24] or with a favorable [21, 22] impact on the course and outcome of disease. For instance, we previously reported a positive impact of a gain of the HER4 gene locus on the outcome of HER2-positive and trastuzumab-treated BC patients [21, 23]. This finding has been later confirmed by others [25]. By way of contrast, the presence of HER4 has been associated with acquired resistance to HER2 inhibitors such as lapatinib [26].

Here, we retrospectively analyzed the impact of HER4 expression on the course and outcome of ER-positive breast cancer patients. To this end, we determined the HER4 mRNA level of 258 ER-positive BC samples by quantitative polymerase chain reaction (qPCR). Subcohorts of pre- and postmenopausal patients were analyzed independently and as a function of treatment with TAM and AIs. These analyses were complemented by in vitro TAM treatment of the strongly ER-positive ZR-75-1 BC cell line. The impact of HER4 on the TAM treatment efficiency was evaluated by siRNA-based HER4 receptor knockdown.

Compared with the cohort of BC patients without or with only low HER4 expression we found poor overall survival of ER-positive and TAM-treated breast cancer patients when the HER4 expression was high. This phenomenon was pronounced and highly significant in postmenopausal women. In contrast to the TAM-treated patients, within the cohort of AI-treated women no impact of HER4 expression on the course and outcome of disease could be observed. In accordance with the analysis on primary tumor samples, the sensitivity of ER- and HER4-positive ZR-75-1 cells to TAM treatment could be significantly enhanced by siRNA-based HER4 knockdown. Taken together, the HER4 expression seems to impair the efficiency of TAM but not AI treatment, even though the predictive value of HER4 in ER-positive BC patients needs to be prospectively evaluated.

## Material and methods

### ER-positive BC database

The pathological diagnostics were performed at the Institute of Pathology at the University of Regensburg (Regensburg, Germany). BC tissues were (immuno)histochemically analyzed based on the estrogen/progesterone receptor, Ki67, and Her2 receptor status and grading and, if applicable, by fluorescence in situ hybridization. Clinicopathological parameters were documented by the Institute of Pathology and the Breast Cancer Center of the University Cancer Center Regensburg. Clinical follow-up was correlated with data from the Tumor Center Regensburg, a population-based regional cancer registry covering a population of more than 2.2 million people including Upper Palatinate and Lower Bavaria. The documentation comprises individual patient data, information on primary diagnosis, treatment regimens, course of disease, and the complete follow-up. Table 1 lists the demographic and clinicopathological data of the patient collective *n* = 258 subjected to this study.

**Table 1:** Demographic data of 258 evaluated hormone receptor-positive patients

Clinicopathological parameter	Premenopausal (*n* = 67)		Postmenopausal (*n* = 191)	
	*n*	%	*n*	%
Tumor stage				
I	32	48	111	58
II	28	42	67	35
III	4	6	8	4
IV	0	0	0	0
Unknown	3	4	5	3
Subtype				
Invasive ductal	56	84	151	79
Invasive lobular	7	10	24	13
Others	4	6	16	8
Grading				
1	5	7	25	13
2	44	66	110	58
3	17	25	52	27
Unknown	1	1	4	2
Lymph node status				
0	30	45	116	61
1	19	28	52	27
2	10	15	13	7
3	6	9	6	3
Unknown	2	3	4	2
Endocrine treatment				
Tamoxifen	43	64	66	35
Aromatase inhibitor	24	36	125	65
Surgery				
Mastectomy	27	40	51	27
Breast-conserving therapy	40	60	140	73
Radiation				
Yes	50	75	151	79
No	7	10	23	12
Unknown	10	15	17	9
Cytotoxicity treatment				
Yes	56	84	85	44
No	1	1	3	2
Unknown	10	15	103	54

### Tissue embedding, processing, and immunohistochemistry

All specimens were acquired from the tissue archive of the Institute of Pathology, University of Regensburg, Germany. The embedding procedure was performed as described elsewhere [27]. Immediately after surgery, the breast tissues were transferred into the formalin fixative (4% formaldehyde, 1% sodium phosphate; SG Planung, Holzkirchen, Germany). The total fixation time was between 12 h (minimum) and 36 h (maximum). The specimens were then subjected to automated dehydration and paraffin immersion. Tissue dehydration was performed by subjecting the tissues to a series of ascending ethanol concentrations (70% for 30 min, 70% for 60 min, 96% for 60 min, 96% for 50 min, 100% for 50 min, and 100% for 90 min), and was completed by incubation in 100% xylene (2 × 50 min). Finally, the tissues were embedded in paraffin with a Shandon Hypercenter XP (2 × 30 min; 2 × 60 min) and 1.5-μm paraffin sections were prepared from the embedded tissue blocks. Specimens were deparaffinized and pretreated by microwave heating for 30 min at 320 W in 0.1 M citrate buffer adjusted to pH 7.3. The immunostaining was automatically performed on a Ventana Nexes autostainer (Ventana, Tucson, USA) by using the streptavidin–biotin peroxidase complex method and 3,3’-diaminobenzidine (DAB) as a chromogen. The autostainer was programmed based on the instructions given by the OptiView DAB detection kit (Ventana). The mouse monoclonal anti-ER antibody clone 6F11 (Leica Microsystems GmbH, Novocastra, Wetzlar, Germany) was used at a dilution of 1:35. The specimens were analyzed by conventional bright field microscopy. ER positivity was rated based on the recommendations given by Remmele and Stegner [3].

### RNA isolation, cDNA synthesis, and HER4-specific real-time qPCR

Four different HER4 isoforms, namely JM-a/CYT1, JM-a/CYT2, JM-b/CYT1, and JM-b/CYT2, resulting from differential splicing have been described while the juxtamembrane (JM)-a variant represents the cleavable form [28]. It has been shown that in BC only the cleavable JM-a isoforms are expressed [23, 29]. Accordingly, we used only HER4/JM-a isoform-specific primers in this study. Base sequences of primers and probes were as follows: forward 5’ CCA CCC ATC CCA TCC AAA-3′, reverse 5’ CCA ATT ACT CCA GCT GCA ATC A-3′, Probe 5’ Fam-ATG GAC GGG CAA TTC CAC TTT ACC A-Dabcyl-3′. We have previously described the qPCR procedure in detail [23]. Briefly, the miRNeasy RNA Isolation Kit (Qiagen, Hilden, Germany) was used to extract RNA from formalin-fixed and paraffin-embedded tissue samples. For synthesis of cDNA, a template of 0.5 μg total RNA was used. According to the manufacturer’s instructions (Transcriptor First Strand cDNA Synthesis Kit; Roche Diagnostics, Mannheim, Germany), the reaction contains random hexamers (Promega, Mannheim, Germany), reverse transcriptase (Promega), dNTP-mixture, and RNAse inhibitor. All reactions were performed in duplicate in the presence and absence of reverse transcriptase. Real-time PCR was performed using fluorescent oligonucleotide LC480 hybridization probes (Metabion, Martinsried, Germany). A calibration standard as well as probes and primers annealing to mRNA of β-actin were used as internal reference and for comparison of successive experiments. PCR was carried out in a final volume of 10 μl containing 2.5 μl cDNA template (1:5 attenuation), 5 μl LC480 Probes Master (Roche), 1 μl probe, and 1.5 μl primers (0.75 μl primer β-actin, 0.75 μl primer target). Probes were labeled with fluorescent reporter dyes FAM (Her4 isoform probes) or LC Red (β-actin probes). Thermal cycling started with the pre-incubation at 95 °C for 10 min. Then amplification was carried out by running 45 cycles, initiated with 30 s at 60 °C followed by 15 s at 95 °C on a LC480 device.

### ZR-75-1 cell line incubation, TAM treatment, and siRNA-based HER4 knockdown

The ZR-75-1 BC cell line was purchased from the American Type Culture Collection (ATCC number CRL-1500, Manassas, VA, USA). For this study, the cell line was authenticated by the Leibniz-Institute “German Collection of Microorganisms and Cell Culture” GmbH (DSMZ, Braunschweig, Germany).

ZR-75-1 cells were cultured in Roswell Park Memorial Institute 1640 medium (RPMI-1640) supplemented with 5% fetal calf serum (FCS) (both from PAN Biotech, Aidenbach, Germany). Cells were commonly seeded at densities of 2 × 10^5^ cells per T75 tissue flask (Greiner Bio-One, Frickenhausen, Germany) and were incubated in a humidified atmosphere containing 5% CO_2_ at 37 °C. Culture medium was refreshed every 2 days. For harvesting, cells were washed with phosphate-buffered saline (PBS; pH 7.4, Biochrom, Berlin, Germany) and were detached from culture flasks by incubating for 3 min at 37 °C in a PBS solution supplemented with 0.05% trypsin and 0.02% ethylenediaminetetraacetic acid.

For the siRNA-mediated HER4 downregulation, 5 × 10^5^ ZR-75-1 cells were seeded in a T25 tissue flask in RPMI-1640 medium supplemented with 5% FCS on day 0. The next day, the medium was removed and 2.5, 2.3, and 2.1 ml (untreated sample/DharmaFECT-treated sample/siRNA sample) fresh RPMI/1% FCS was added, respectively. The transfection mix was prepared by incorporation of 10 μl DharmaFECT (Dharmacon, Lafayette, CO, USA) with 190 μl Opti-MEM (Invitrogen, Karlsruhe, Germany) in tube 1 and 12.5 μl of 10 μM anti-HER4 siRNA (L-003128-00-0005 ON-TARGETplus SMARTpool Human ERBB4 2066, Dharmacon) and 187.5 μl Opti-MEM in tube 2. For control purposes we exposed the cells to non-targeting siRNA (D-001810-10-05 ON-TARGETplus Non-targeting Pool; Horizon Discovery Ltd., CA, USA) which is expected to cause no effect on receptor expression and cell proliferation. After 5 min incubation at room temperature the contents of tube 1 and tube 2 were pooled and subsequently thoroughly mixed. After a further incubation step of 20 min at room temperature the 400-μl transfection mix was added per flask to give a final siRNA concentration of 50 nM. From day 2 on, the cells were treated with 5 μM TAM for 96 h (Sigma-Aldrich Chemie GmbH, Deisenhofen, Germany).

### Western blotting

Treated and untreated ZR-75-1 cells were lysed for total protein analysis in cell-lysis buffer (Cell Signaling, Danvers, MA, USA) supplemented with Halt™ Protease and Phosphatase Inhibitor Cocktail (Thermo Fisher Scientific, Bremen, Germany). After calculating the protein concentration with the Pierce BCA protein assay kit (ThermoFisher), 25 μg total protein per lane were separated in 7.5% SDS-PAGE under reducing conditions (mercaptoethanol) and blotted onto polyvinylidene difluoride (PVDF) membranes. Membranes were blocked with Tris-buffered saline supplemented with 5% low-fat milk and 2% Tween for 2 h and then incubated overnight at 4 °C using the following primary antibodies: rabbit anti-human HER1 (D38B1; 1:1000), rabbit anti-human HER2 (29D8; 1:4000), rabbit anti-human HER3 (D22C5; 1:200), rabbit anti-human HER4/ErbB4 (111B2; 1:1000) (all from Cell Signaling Technology), rabbit anti-human β-actin (A2066; 1:5000, Sigma-Aldrich Chemie GmbH), and mouse anti-estrogen receptor (NCL-L-ER-6F11; 1:500, Leica Microsystems). The next day, after washing the membrane, incubation with secondary antibodies (goat anti-rabbit 7074 HRP-conjugated and horse anti-mouse (7076) HRP-conjugated; both 1:2000, and both from Cell Signaling Technology) was performed for 1 h at room temperature. The blots were visualized using the chemiluminescent Western blotting detection system (GE Healthcare, Amersham, UK) and analyzed by ImageQuant LAS 4000 mini-imager (GE Healthcare).

### Proliferation assessment by flow cytometry

BrdU/Hoechst quenching measurements (i.e., the assessment of G0-phase fraction) were performed as described previously [30]. This approach is based on continuous labeling of cells in vitro with BrdU which is incorporated into the DNA instead of thymidine during the cell cycle S phase. BrdU incorporation results in quenching of Hoechst 33258 but not of propidium iodide upon DNA double staining. Thus, actively proliferating cells and quiescent (i.e., G0 phase) cells can be differentiated and separately quantified. For flow cytometric cell analyses, 5 × 10^5^ cells were seeded on day 0 into T25 culture flasks and were incubated for 7 days. At day 4, 120 μM BrdU was added to the culture flasks and cells were incubated in the presence of BrdU for an additional 72 h. To minimize potential disturbance in the nucleotide pathway due to BrdU treatment, the medium was also supplemented with half-equimolar 2’deoxycytidine. After detachment the cells were stored at −20 °C at a concentration of 10^6^ cells/ml in freezing medium (RPMI-1640 medium + 10% FCS + 10% dimethyl sulfoxide (DMSO)) until flow cytometric analysis. For cell staining, thawed cells were washed twice with 2 ml ice-cold DNA-staining buffer (100 mM Tris-HCl, pH 7.4, 154 mM NaCl, 1 mM CaCl_2_, 0.5 mM MgCl_2_, 0.1% IGEPAL CA-630 (Nonylphenylpolyethylenglycol), 0.2% bovine serum albumin (BSA)); 5 × 10^5^ cells were resuspended in 1 ml buffer supplemented with 40 g/ml (2–4 units/ml) RNase and 1.2 μg/ml Hoechst 33258 (Sigma-Aldrich) and incubated for 15 min at 37 °C. Cellular DNA content was stained with propidium iodide (1.5 μg/ml) for 15 min on ice. Samples were passed through a 70-μm nylon mesh to remove cell aggregates prior to flow cytometric analysis. Flow cytometric measurements were performed on a FACSCanto II flow cytometer (BD Biosciences, San Jose, CA) equipped with a blue (488 nm), red (633 nm), and violet (405 nm) laser and a standard optical configuration. Sample measurements and data analysis were performed with FACSDiva Software v7.0 (BD Biosciences), and 50,000 events/sample were collected. As described previously in detail, the G0 cell fraction was calculated by taking into account the fraction of cells that had divided once, twice, or three times within the period of observation [31].

### Statistical analyses

Overall survival (OS) was calculated from the date of diagnosis to the date of death of any cause. Patients who survived were classified as censored cases at the latest date they were confirmed to be alive. Disease-free survival (DFS) was calculated as the period of time after (successful) primary treatment without any evidence of cancer-related signs, symptoms, or death. Patients without any cancerous disease and being alive were classified as censored cases at the latest date they were confirmed to be disease free and alive. Maximum follow-up time was set to 10 years. Patients with a longer follow-up were classified as censored cases after 10 years. The impact of HER4 expression on DFS and OS was calculated for all patients and subcohorts (i.e., pre- versus postmenopausal, TAM- versus AI-treated patients). Survival curves were estimated using the Kaplan–Meier method and hazard ratios (HRs) and corresponding 95% confidence intervals (Cis) were calculated by Cox proportional hazards regression models for the univariate as well as for the multivariable models. An optimal cut-off for HER4 values for predicting OS and DFS was estimated based on log-rank statistics. All reported *p* values were two-sided. A *p* value lower than 0.05 was considered to indicate a significant difference. All statistical analyses were performed using R version 3.3.3 (The R Foundation for Statistical Computing) or GraphPad Prism (Ver. 6, GraphPad Software, La Jolla, CA, USA).

## Results

### Considering the entire collective HER4 expression has no significant impact on the outcome of disease

No correlation of HER4 expression (continuously or dichotomized) with the DFS or the OS could be found when all patients (i.e., without regard to the treatment regimen and age) were included into the analysis (Table 2).

**Table 2:** Impact of HER4 expression on the overall survival (OS) and disease-free survival (DFS) in all patients (independent of menopausal status and treatment)

Predictor	OS				DFS			
	HR	95% CI		*p* value	HR	95% CI		*p* value
HER4 continuous	1.08	0.87	1.35	0.474	1.00	0.80	1.25	0.993
HER4-positive (ref. no HER4 expression)	0.90	0.49	1.66	0.736	0.91	0.54	1.55	0.733

*CI* confidence interval, *HR* hazard ratio

### HER4 expression has a significant impact on the outcome of TAM-treated but not on the outcome of AI-treated patients

When considering TAM-treated patients, a significant impact of HER4 expression on the OS was found (HR = 1.28 HER4 continuous; HR 3.22, HER4 ≥ 1, respectively; Table 3). Further analyses identified a better cut-off for the HER4 expression of the TAM-treated group, with HER4 < 1 (HER4-negative) versus HER4 ≥ 1 (HER4-positive). In contrast to the TAM-treated cohort, in the AI-treated subpopulation no correlation of HER4 expression to the OS (HR = 0.86 and 0.68, respectively) or DFS (HR = 0.85 and 0.72, respectively) was seen (Table 3).

**Table 3:** Impact of HER4 expression on the overall survival (OS) and disease-free survival (DFS) in patient subcohorts stratified by treatment (TAM or AI)

Subgroup	Predictor	OS				DFS			
		HR	95% CI		*p* value	HR	95% CI		*p* value
TAM treated	HER4 continuous	**1.28**	**1.01**	**1.62**	**0.040**	1.16	0.89	1.52	0.270
	HER4 expression (ref. no HER4 expression)	1.63	0.52	5.06	0.399	1.23	0.54	2.84	0.622
	HER4 ≥ 1 (ref. HER4 < 1)	**3.22**	**1.17**	**8.86**	**0.024**	1.36	0.63	2.97	0.436
Al treated	HER4 continuous	0.86	0.60	1.22	0.393	0.85	0.61	1.18	0.338
	HER4 expression (ref. no HER4 expression)	0.68	0.31	1.50	0.339	0.72	0.36	1.44	0.352

Significant values are shows in bold typeface

*AI* aromatase inhibitor, *CI* confidence interval, *HR* hazard ratio, *TAM* tamoxifen

Further stratification of the TAM-treated group based on the pre- and postmenopausal status (< 46 years vs. ≥ 46 years) revealed that HER4 expression had a significant impact on the OS (HR =1.43, HER4 continuous; HR =4.98, HER4 ≥ 1) and DFS (HR = 1.81, HER4 continuous) only in postmenopausal but not in premenopausal women (Table 4).

**Table 4:** Impact of HER4 expression on the overall survival (OS) and disease-free survival (DFS) in tamoxifen-treated patients stratified by menopausal status

Strata	Predictor	OS				DFS			
		HR	95% CI		*p* value	HR	95% CI		*p* value
Premenopausal (< 46 years)	HER4 continuous	1.06	0.59	1.89	0.845	0.77	0.43	1.38	0.380
	HER4 ≥ 1 (ref. HER4 < 1)	1.51	0.25	9.01	0.655	0.56	0.15	2.03	0.377
Postmenopausal (≥ 46 years	HER4 continuous	**1.43**	**1.09**	**1.86**	**0.009**	**1.81**	**1.25**	**2.63**	**0.002**
	HER4 ≥ 1 (ref. HER4 < 1)	**4.98**	**1.32**	**18.80**	**0.018**	2.94	0.96	9.00	0.059

Significant values are shows in bold typeface

*CI* confidence interval, *HR* hazard ratio

**Fig. 1:**
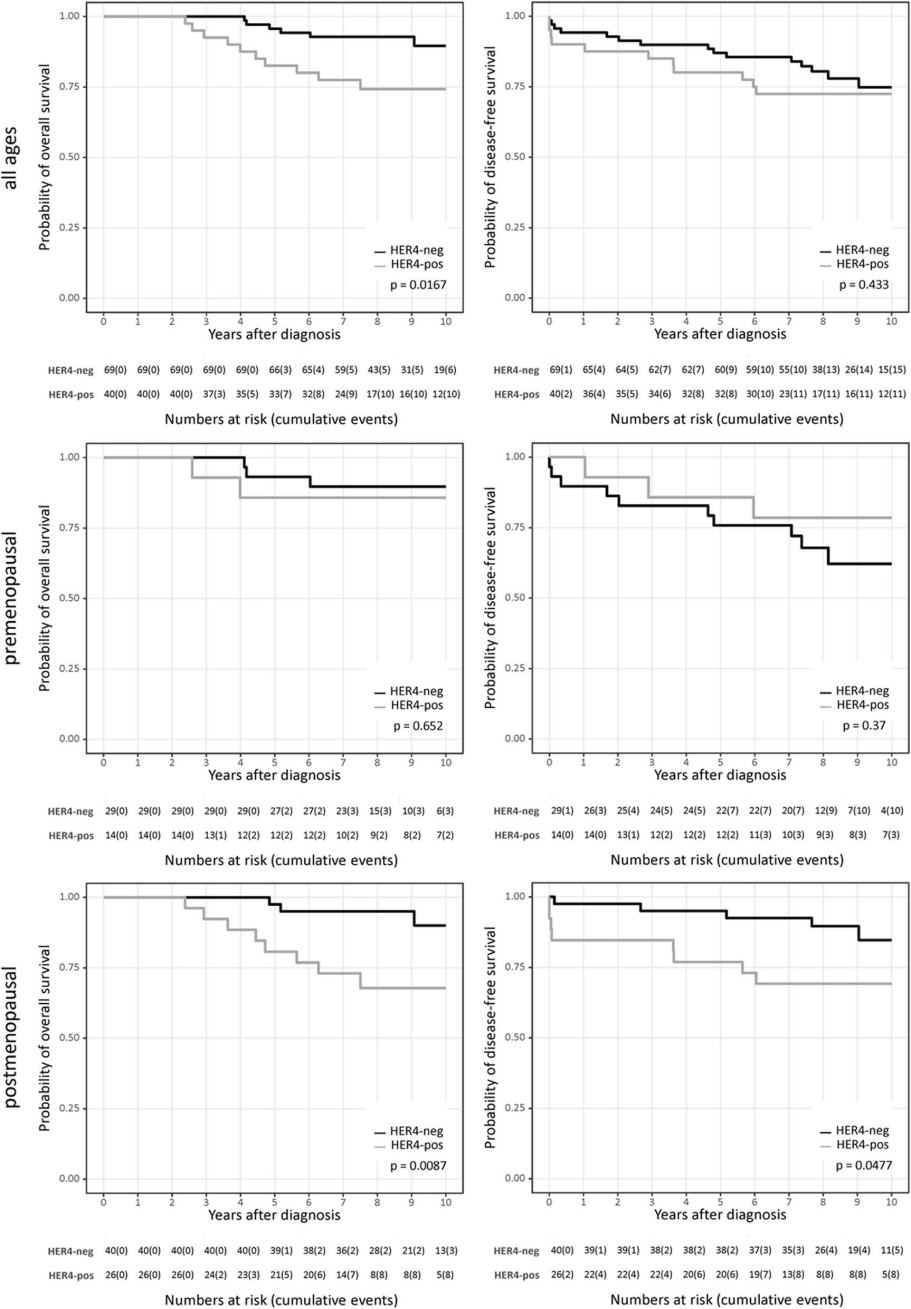
Disease outcome of TAM-treated ER-positive breast cancer patients. OS and DFS are displayed for all patients (top row), only premenopausal (< 46 years; middle row), and only postmenopausal (≥ 46 years, bottom row) breast cancer patients treated with TAM. The *p* values were calculated using the log-rank test (Mantel–Cox) and are indicated in each graph

Kaplan–Meier survival curves confirmed that the OS of TAM-treated patients was significantly (*p* = 0.0167) improved in HER4-negative BC patients, which was independent of their menopausal status (Fig. 1, left column). No significant difference (*p* = 0.433) was detectable with respect to the DFS (Fig. 1, right column). Classification by age revealed no significant differences for DFS (*p* = 0.37) or OS (*p* = 0.652) in TAM-treated premenopausal (< 46 years of age) patients (Fig. 1, middle row). However, the OS (*p* = 0.0087) was significantly impaired in postmenopausal HER4-positive patients (≥ 46 years of age; Fig. 1, bottom row). The DFS of postmenopausal and TAM-treated women tends to be significantly better in case of HER4-negative BC compared with HER4-positive BC (*p* = 0.0477; Fig. 1, bottom row).

**Fig. 2:**
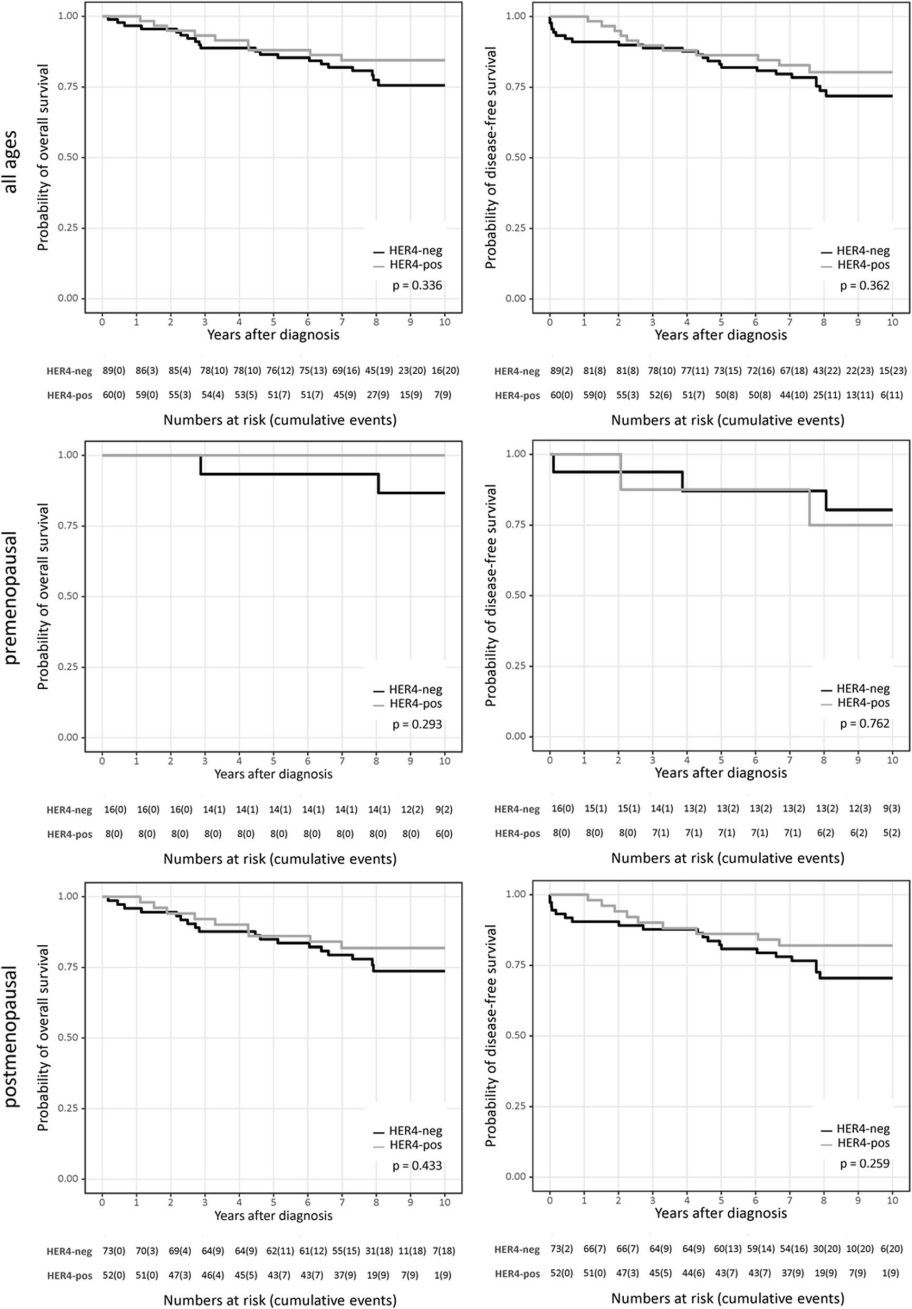
Disease outcome of AI-treated ER-positive breast cancer patients. OS and DFS are displayed for all patients (top row), premenopausal (< 46 years; middle row), and postmenopausal (≥ 46 years, bottom row) breast cancer patients treated with AI. The *p* values were calculated using the log-rank test (Mantel–Cox) and are indicated in each graph

Patients treated with AIs did not display any significant HER4 dependency with respect to OS and DFS, neither in pre- nor in postmenopausal patients (all *p* values greater than 0.05, Fig. 2). However, a trend towards an improved (rather than to an impaired) course and outcome of disease in HER4-positive patients compared with women with HER4-negative tumors became apparent.

To assess if HER4 is an independent predictor for OS and DFS within the TAM-treated postmenopausal women, a multivariable Cox regression model was applied (Table 5). Due to the limited number of events only two further covariables were added to the model. Based on clinical relevance and statistical significance, patient age and tumor staging (pT) were selected. In the adjusted models, HER4 as a continuous variable remains a significant predictor for both OS (*p* = 0.035, HER4 continuous) and DFS (*p* = 0.003, HER4 continuous).

**Table 5:** Multivariable Cox regression analysis of tamoxifen-treated postmenopausal women on the overall survival (OS) and disease-free survival (DFS) including HER4, patient age, and tumor stage

	OS (*n* = 64, events = 12)				DFS (*n* = 64, events = 12)			
	HR	95% CI		*p* value	HR	95% CI		*p* value
Model 1								
HER4 continuous	**1.41**	**1.02**	**1.95**	**0.035**	**2.34**	**1.34**	**4.06**	**0.003**
Age	1.14	1.07	1.21	< 0.001	1.11	1.05	1.18	< 0.001
pT stage (ref. pT1)	4.98	1.11	22.37	0.036	4.01	1.10	14.64	0.035
Model 2								
HER4 ≥ 1 (ref. HER4 < 1)	**3.89**	**0.92**	**16.37**	**0.064**	2.38	0.71	8.00	0.161
Age	1.12	1.05	1.18	< 0.001	1.09	1.04	1.15	0.001
pT2 or higher (ref. pT1)	6.68	1.60	27.93	0.009	3.80	1.13	12.80	0.031

Significant values that refer to HER4 are shown in bold typeface

*CI* confidence interval, *HR* hazard ratio, *pT* tumor stage

### HER4 downregulation enhanced efficiency of TAM treatment in vitro

**Fig. 3:**
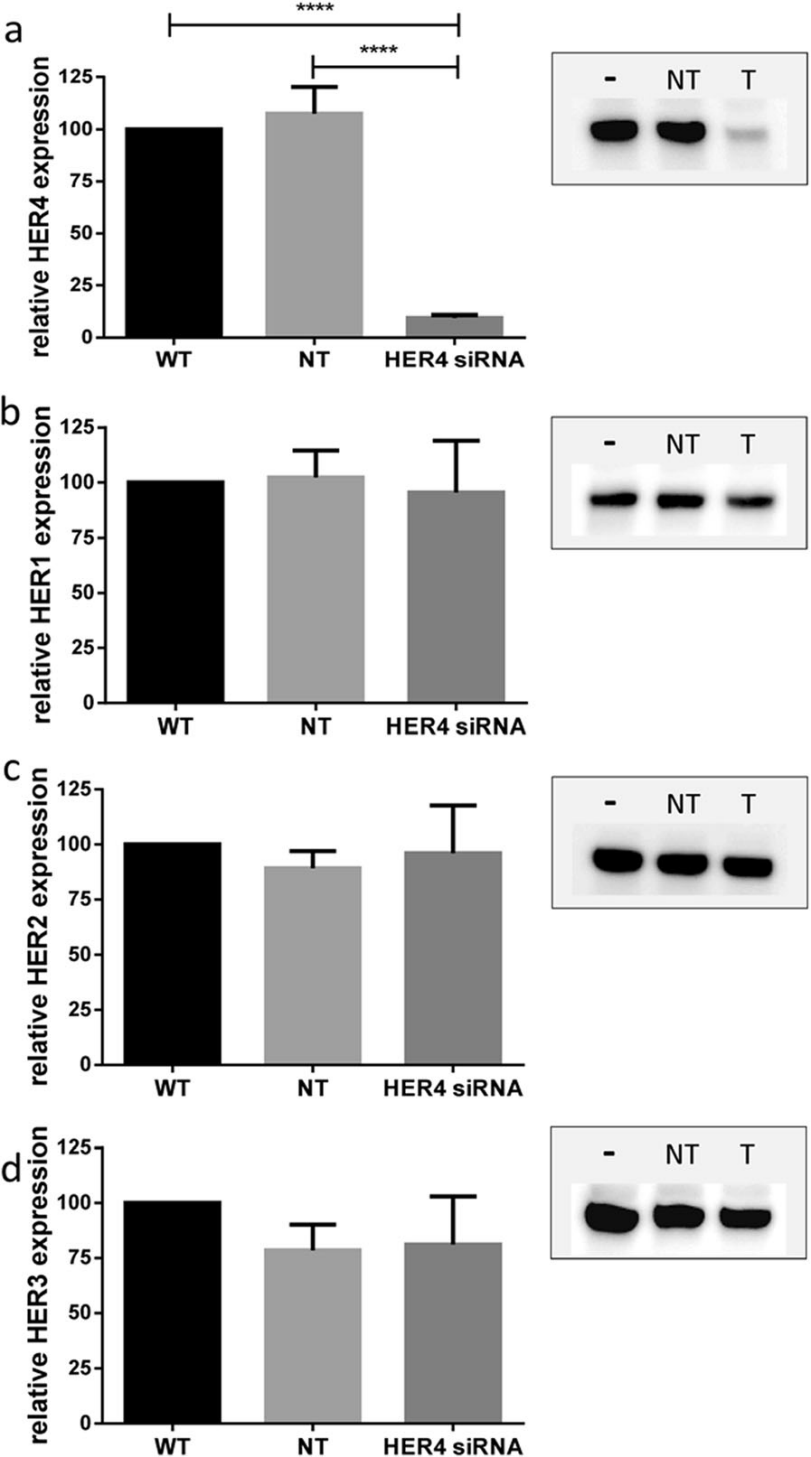
Reduced HER4 protein expression in siRNA-treated ZR-75-1 breast cancer cell line. Western blot analyses of HER4 (**a**), HER1 (**b**), HER2 (**c**), and HER3 (**d**) are shown for cell lysates gained from untreated ZR-75-1 control cells (–), cells treated with non-targeting siRNA (NT), and ZR-75-1 cells treated with targeting anti HER4/siRNA (T). One representative blot is shown, respectively, and repeated experiments (*n* = 3) are summarized in the corresponding bar charts. *P* values are calculated using one-way ANOVA and Tukey’s multiple comparisons test (****p < 0.0001). WT wild-type

**Fig. 4:**
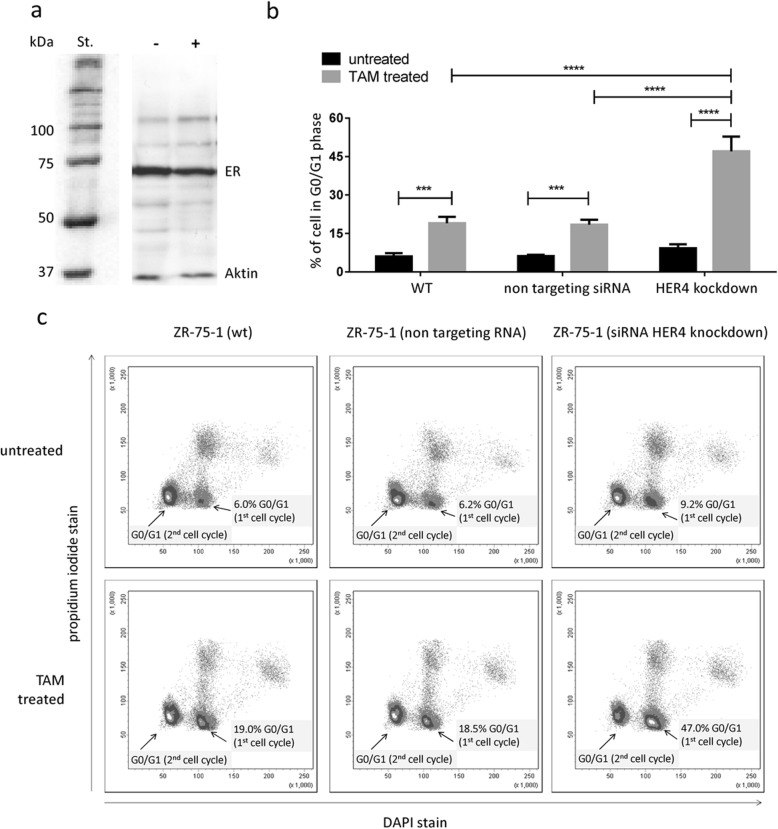
HER4 siRNA knockdown enhances the tamoxifen (TAM)-induced G0/G1 fraction. **a** The expression of the estrogen receptor (ER) in ZR-75-1 wild-type cells is exemplarily shown by Western blot. St. represents the protein standard and – and + refer to the samples without and with anti-HER4 siRNA treatment, respectively. The molecular weight of the ER is about 66 kDa. The ER expression was not affected by an anti-HER4 siRNA treatment. **b** Percentage of G0/G1 phase (quiescent) cells in wild-type (WT), non-targeting siRNA control, and HER4 knockdown ZR-75-1 BC cells with or without TAM treatment is shown (*n* = 3; mean ± SD). *P* values are calculated using two-way ANOVA and Sidak’s multiple comparisons test (***p < 0.001; ****p < 0.0001). **c** Example density plots from flow cytometric analyses (BrdU/Hoechst quenching assay) are displayed. The size of the G0/G1 fraction of the first cell cycle is indicated, respectively

HER4 knockdown in ER-positive ZR-75-1 breast cancer cells resulted in about 90% reduced HER4 protein levels (Fig. 3a). Off-target effects on the other members of the HER receptor family (i.e., HER1, HER2, and HER3) could be excluded (Fig. 3b–d). The unaffected ER expression in ZR-75-1 cells is exemplarily shown in Fig. 4a. BrdU/Hoechst quenching assay was applied to quantify the different TAM efficiencies in wild-type (WT) and HER4 knockdown cells. This technique allows the quantitative assessment of proliferating and resting cells. Cells which stop proliferation are considered to become quiescent and to enter the G0/G1 resting phase. Repeated measurements revealed a significantly increased fraction of G0-phase cells in the presence of TAM treatment compared with untreated cells (Fig. 4b). This observation applies to both WT (*p* < 0.001) cells and cells treated with non-targeting siRNA (*p* < 0.001). However, TAM treatment efficiency is significantly enhanced in ZR-75-1 cells upon HER4 receptor knockdown compared with ZR-75-1 cells with regular HER4 expression (on average 19% versus 47.0%, *p* < 0.0001). Flow cytometric example measurements are shown in Fig. 4c. An increased fraction of G0/G1 resting cells upon TAM treatment was measured in ZR-75-1 wild-type cells (6% in untreated vs. 19% in treated cells). This effect was significantly enhanced upon HER4 knockdown (9.2% in untreated vs. 47% in treated cells; *p* = 0.0001). The quiescent cells were not able to proceed with cycling within the period of treatment/observation and are blocked in G0/G1 of the first cycle.

## Discussion

For about four decades, TAM (which binds to and antagonizes the ER) has been the mainstay of endocrine therapy in both early and advanced breast cancer patients. Although the hormone treatment has brought significant benefit to hormone receptor-positive BC patients, up to 50% of patients with advanced disease do not respond to first-line treatment but show de novo resistance. Another significant cohort of advanced BC patients in the adjuvant setting acquire resistance while treated with TAM and later on develop tumor relapse [20]. Mechanisms that contribute to or directly cause resistance to the TAM treatment are manifold [19], although receptor tyrosine kinases (e.g., HER2) are frequently involved. In order to evade TAM resistance, AIs that inhibit the endogenous synthesis of the native ER activating ligand estrogen can be administered sequentially with TAM [20] or as first-line treatment, especially for postmenopausal women. Here, we retrospectively analyzed the impact of HER4 on the course and outcome of TAM- or AI-treated patients with ER-positive BC. In addition, we evaluated the TAM treatment efficiency of ER-positive ZR-75-1 BC cells in vitro as a function of HER4 receptor expression.

We found a significant unfavorable effect of HER4 in patients treated with TAM but not in women treated with AIs. A detailed analysis of sample subcohorts further disclosed a strong and significant impact in postmenopausal but not in premenopausal patients. In vitro analyses revealed that the proliferation of markedly ER-positive ZR-75-1 cells was inhibited when exposed to TAM. However, the treatment efficiency was significantly enhanced upon siRNA-based HER4 knockdown.

**Fig. 5:**
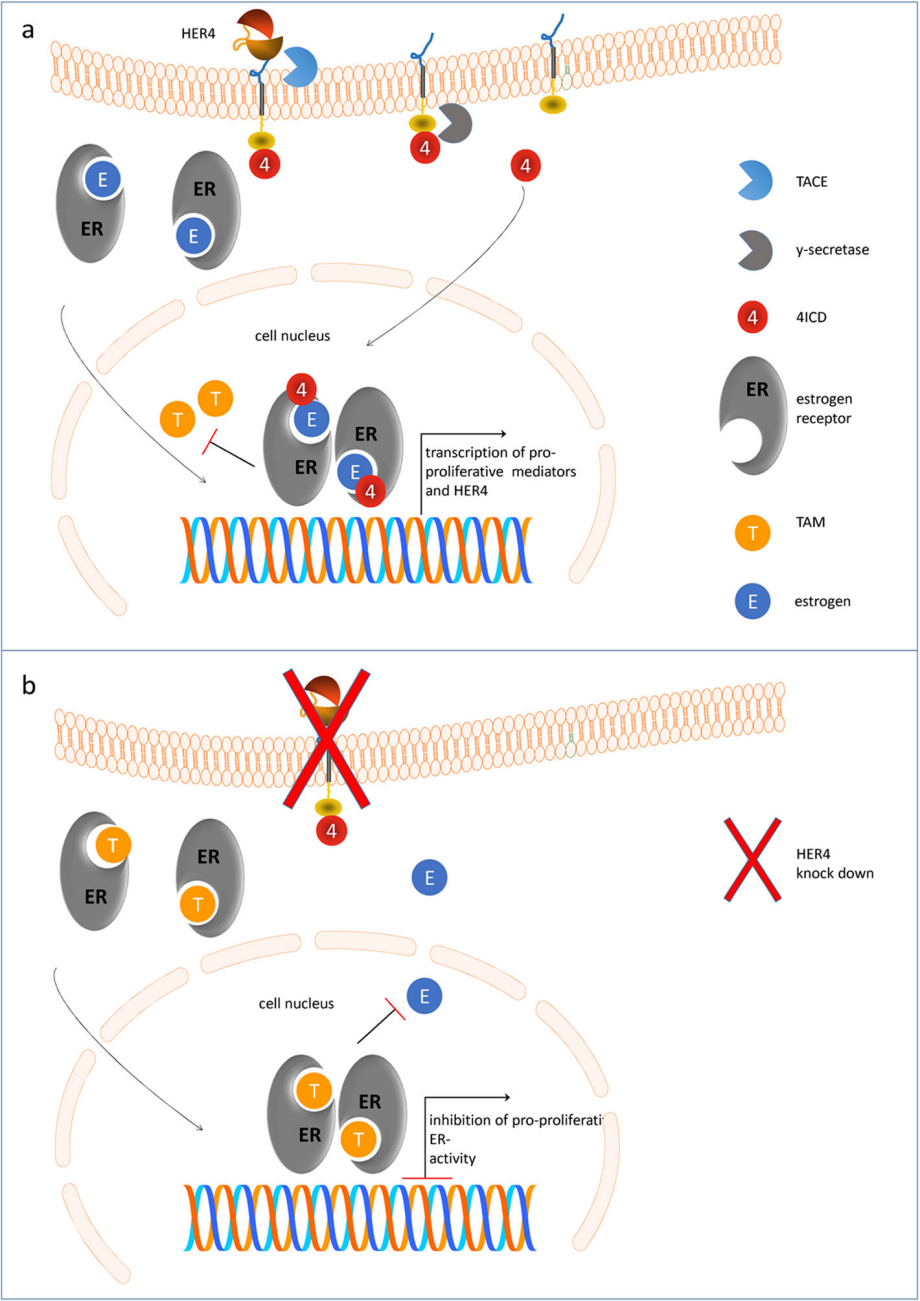
Suggested mechanism of estrogen receptor (ER) action in the presence and absence of HER4. **a** HER4 can be processed by a two-step proteolytic activation. First tumor necrosis factor α converting enzyme (TACE) cleaves the extracellular domain, and subsequently y-secretase cleaves the intracellular domain of HER4 (4ICD) which is released into inner cell compartments. If translocated into the nucleus, 4ICD as an ER co-activator enhances the pro-proliferative effects of estrogen. Within an autoloop, 4ICD also enhances the transcription of HER4 itself. **b** HER4 receptor knockdown eliminates the co-stimulatory activity of 4ICD. As a consequence, appropriately dosed tamoxifen (TAM) can competitively replace the estrogen and binds to the ER. As a result, pro-proliferative activity of the ER is inhibited

Apparently, HER4 impedes the efficiency of TAM but not AI treatments. The unfavorable impact of HER4 on the outcome of TAM- but not AI-treated patients suggests a direct interaction of HER4 with the ER. A possible explanation is the ER-stimulating activity of the intracellular HER4 domain when translocated into the tumor cell nucleus [32, 33] (Fig. 5). A nuclear localization can be explained by a two-step intramembrane proteolysis of the HER4 receptor [34]. First, a metalloproteinase called tumor necrosis factor α converting enzyme (TACE) can cleave and release the HER4 ectodomain. Second, this proteolysis can be followed by an intracellular cleavage performed by γ-secretase that releases an intracellular domain (4ICD) into inner cell compartments. Depending upon specific molecular interactions [35], the 4ICD either translocates to the nucleus or remains in the cytosol where mitochondrial accumulation has been observed. As a protein with pronounced BCL-2 homology-3, the 4ICD can interact with pro-apoptotic molecules located in the mitochondrial membrane and initiate apoptotic cell death by cytochrome-c release [33, 36]. In contrast, when transferred into the nucleus, 4ICD can work as a co-activator of the estrogen receptor and - in the presence of exogenous estrogen - contribute to enhanced (tumor) cell growth [32] and thereby possibly account for an unfavorable disease course [37, 38]. The subcellular localization might also be determined by a differential expression of intracellular isoform domains [33, 34, 38–40]. Even if only the juxtamembranous cleavable isoform JM-a (but never JM-b) is expressed in BC [28, 29, 39, 40], two different cytoplasmatic domains (i.e., CYT1 and CYT2) potentially occur also in the sample cohort of this study. Notably, CYT1 and CYT2 domains have been shown to interact with different intracellular molecules that are involved in cell compartment (cytoplasm vs. nucleus)-specific 4ICD routing [41]. In this study, we did not differentiate the two HER4-specific intracellular domains; however, a preferred routing to the cell nucleus might have contributed to the unfavorable impact of HER4 expression on ER-positive/TAM-treated patients. The assumed mechanisms of ER action in the presence and absence of HER4/4ICD are illustrated in Fig. 5. The two-step proteolytic activation of HER4 by TACE and y-secretase [29] causes the release of 4ICD into inner cell compartments. If translocated into the nucleus, 4ICD, as an ER co-activator, enhances the pro-proliferative effect of estrogen. Within an autoloop, 4ICD also enhances the transcription of HER4 itself. In contrast, HER4 receptor knockdown eliminates the co-stimulatory activity of 4ICD. As a consequence, appropriately dosed TAM can competitively replace the estrogen and binds to the ER. As a result, pro-proliferative activity of the ER is inhibited.

We previously associated the presence of HER4 with an improved outcome in BC patients [21, 22]. In contrast to former studies, however, we herein explicitly explored hormone receptor (i.e., ER)-positive BCs, and HER4 might mediate different effects in different BC subtypes. Indeed, it has been experimentally shown that TAM disrupts an estrogen-driven interaction between ER and 4ICD while promoting mitochondrial accumulation of the 4ICD/BH3-only protein [36]. Accordingly, it seems plausible that a 4ICD/ER interaction impairs TAM binding and reduces treatment efficiency both in vivo and in vitro. The increased sensitivity of ZR-75-1 cells to TAM treatment on HER4 knockdown supports the assumption that the HER4/4ICD directly interferes with the TAM-ER binding. Nevertheless, other mechanisms that underlie HER4/4ICD-mediated reduced TAM efficiency cannot be excluded.

An ER/4ICD interaction would explain the reduced efficiency of TAM treatment in the presence of HER4/4ICD and the improved outcome of patients with ER^+^/HER4^low^ tumors compared with patients who suffer from ER^+^/HER4^high^ tumors. Moreover, it would also be compatible with the lack of impact of HER4 in AI-treated patients. Since AIs do not affect the ER function but inhibit the synthesis of the ER ligand, a direct ER/4ICD interaction would neither be affected by an administration of AIs, nor would the AI treatment efficiency be affected by the presence of HER4 (i.e., the 4ICD/ER crosstalk).

In AI-treated patients we found a trend towards an improved (rather than to an impaired) course and outcome of disease in HER4-positive patients. Notably, this trend is in reverse to the negative significant effect of HER4 in TAM-treated patients and might be explained by the absence (or reduced systemic level) of estrogen in AI-treated women. A reduced presence of ER might entail a pronounced accumulation of 4ICD in the cytoplasm where it induces a rather tumor-suppressive effect by a pro-apoptotic (i.e., favorable) activity [33, 42, 43].

Notably, stratified analyses revealed a significant impact of HER4 in ER-positive and TAM-treated BC patients for postmenopausal but not for premenopausal women. This finding might to some extent be explained by different age-related estrogen synthesis. In premenopausal women estrogens are mainly synthesized in the ovaries, whereas in postmenopausal women the final synthesis occurs in peripheral tissues such as mesenchymal cells from adipose tissue (including the breast), bones, muscle, or brain. Important for the final synthesis is aromatase which can be detected in non-malignant adipose tissue but also in breast tumors [43]. Indeed, the estrogen level in the peripheral blood in premenopausal women is higher compared with postmenopausal women [44]. However, the tissue concentration of estrogen sulfate, sulfatase, and aromatase activities was significantly higher in postmenopausal women [45]. In addition, significantly higher estrogen concentrations could be even found in malignant versus normal tissues [46, 47]. Moreover, the ER level affects the estradiol levels found in tissues and can cause a significant (up to 8-fold) increased estradiol level in ER-positive tumor tissue [48]. Hence, the local estrogen synthesis is increased especially in ER-positive tumors. Provided that the anti-estrogen TAM is mainly effective in the (local) presence of estradiol it appears plausible that an ER-TAM-HER4/4ICD interaction is relevant in particular in postmenopausal women. Thus, a reduced efficiency of TAM treatment in elderly BC patients becomes mainly apparent in the presence of HER4. This finding might to some extent also be due to the larger sample cohort of postmenopausal women compared with the number premenopausal patients.

The finding of impaired TAM treatment efficiency in the presence of HER4 might have clinical implications and might promote alternate therapeutic strategies, particularly in postmenopausal women. Analogously to ER and HER2 testing, the BC diagnostics could be extended by the evaluation of HER4 expression. If the TAM treatment efficiency is low in an ER/HER4 double-positive tumor one might preferably switch to an AI treatment. Alternatively, combined ER/HER4 receptor targeting might restore TAM sensitivity or even enhance the endocrine treatment efficiency. Anti-HER4 targeting can be performed based on different strategies. On the one hand, an HER4 JM-a type-specific anti-HER4 monoclonal antibody, called Ab1479, has been reported to block HER4 cleavage in BC cells and to suppress BC cell growth in vivo and in vitro [34, 49]. A systematic clinical trial on ER/HER4 double-positive BC could potentially result in clinical approval of Ab1479 for the treatment of this BC entity. Alternatively, pan-HER-receptor inhibitors, e.g., afatinib or neratinib, could be administered in combination with an anti-estrogen [50, 51]. By using the latter strategy not only HER4 but also the potentially expressed HER2 receptor, which is frequently involved in TAM resistance, would be targeted at the same time. After all, in addition to immunohistochemistry and in-situ hybridization, a number of quantitative and multiplexed HER1–4 analytics became available to quantify HER1–4 receptors and categorize the patients for individualized treatments [21, 52, 53]. However, all the suggested strategies require prospective clinical testing and approval in advance.

## Conclusion

Here we provide evidence for the HER4 receptor as a new predictive marker for the sensitivity of ER-positive BC to TAM treatment, especially in postmenopausal patients. Dual ER/HER4 targeting might improve the treatment efficiency of hormone receptor-positive BC but needs to be prospectively evaluated in an appropriate preclinical and clinical setting.

## Abbreviations

4ICD: Intracellular domain of HER4
AI: Aromatase inhibitor
BC: Breast cancer
CI: Confidence interval
CYT: Cytoplasmatic domain
DFS: Disease-free survival
ER: Estrogen receptor
HER: Human epidermal growth factor-related receptor
HR: Hazard ratio
JM: Juxtamembrane
OS: Overall survival
TACE: Tumor necrosis factor α converting enzyme
TAM: Tamoxifen
WT: Wild-type
